# Supplementary material for: Are carbon emissions trading and green financial instruments synergistic? -Comprehensive quantitative research based on content analysis
Source: PLoS One. 2024 Mar 7;19(3):e0298601. doi: 10.1371/journal.pone.0298601 (PMC10919601; doi:10.1371/journal.pone.0298601)
Supplement: S1 Data — (ZIP) [file pone.0298601.s001.zip › Data/地级市Z_F工作报告.zip!/║■─╧/╘└╤⌠╩╨/└┤╫╘╣1⁄2╓┌║┼í╛┬φ┐╦╩2╛▌═°í┐_2012.docx]

各位代表：

　　我代表市人民政府，向大会报告政府工作，请予审议，并请各位政协委员和其他列席人员提出意见。

　　一、2011年实现 “十二五”良好开局

　　2011年是实施“十二五”规划的第一年，全市上下在中共岳阳市委的坚强领导下，努力践行民本岳阳执政和发展理念，以“科学发展、富民强市”为主题，以“转型升级、更大更强”为主线，围绕“四化两型”和“五市一极”建设，团结一心，奋力拼搏，完成了市六届人大四次会议确定的年度目标任务，各项工作取得新的成绩，实现了“十二五”开门红。

　　(一)科学应对挑战，保持了经济平稳较快增长。面对发展中凸显的诸多挑战，我们科学应对，战胜严重的春夏连旱和局部地区300年一遇的特大山洪泥石流等自然灾害，努力化解资金、土地、能源等生产要素趋紧的制约，经济发展呈现良好态势。预计全市实现地区生产总值1900亿元，同比增长13.8%;完成财政总收入183.8亿元，增长31.7%;完成固定资产投资858亿元，增长35%;实现社会消费品零售总额595.7亿元，增长17.5%;三次产业结构调整为 13.4:55.8:30.8;非公经济增加值占GDP比重达到58.5%;万元GDP能耗同比下降3%;城镇居民人均可支配收入19558元，增长13.1%;农民人均纯收入7186元，增长20%。国家统计局评定我市名列全国城市综合实力第57位。汨罗、华容、岳阳县进入全省县域经济前20强，君山获评全省经济发展先进县(市)区。

　　新型工业化加速推进。规模工业企业总数达到1345家，预计规模工业增加值增长20%。工业主导作用进一步增强，对经济增长的贡献率达到62.3%，工业企业实缴税收达到100亿元。石化产业成为我市首个千亿产业，总产值达1020亿元。百亿园区达到7个，园区工业增加值占全市规模工业增加值比重41.8%。非公规模工业企业增加值增长23%。平江工业园、云溪工业园获评“国家新型工业化产业示范基地”。科技创新能力进一步增强，全市获国家、省级科技进步奖16项，授权专利850件，新增高新技术及其产品企业40家，总数达到237家，高新技术产值增幅达32%。临湘被定为“湖南省知识产权试点县(市)”。

　　农业现代化扎实推进。粮食生产又获丰收，预计总产量达307.7万吨;出栏生猪753万头;水产品产量达38万吨，继续保持全省第一。规模农产品加工企业达到291家，完成增加值142.7亿元，增长22%;新增巴陵油脂、九鼎科技2家国家级农业产业化重点龙头企业;新增插旗、长康、现代美、山润、华康5个涉农中国驰名商标。新增农民专业示范合作社185个，总数达574个。我市被授予“中国黄茶之乡”称号;华容获评“全国农业标准化示范县”;湘阴、华容荣获“全国粮食生产先进县”称号;君山获批“中国绿色食品建设示范基地”;屈原国家级现代农业示范区建设稳步实施。

　　第三产业加快发展。预计第三产业完成增加值585亿元，同比增长15%。航运物流业日益趋旺，实现1.2万吨散货船舶和5000吨集装箱船舶常年通航，全市集装箱吞吐量达16万标箱，增长33.3%，物流业增加值占GDP比重达8.2%。商贸流通更加活跃，城乡流通网络进一步完善，“万村千乡”农家店总数居全省第一。旅游业不断升温，全年接待国内外游客1581.7万人次，旅游总收入131.3亿元，增长25.6%。岳阳楼-君山岛晋升国家5A级旅游景区，洞庭湖旅游度假区挂牌，君山获批“全国休闲农业与乡村旅游示范区”，平江获评“全国红色旅游先进县”。房地产业健康发展，完成投资100亿元，商品房销售面积增长38%。金融业平稳运行，全市金融机构新增存款余额163.5亿元，新增贷款余额80.3亿元。引进国家开发银行在汨罗设立村镇银行，组建平江博创兴业小额贷款公司，设立湖南碧城创业投资基金和湖南城陵矶临港产业新区创业投资基金，全市保险公司发展到33家。凯美特气在深交所中小板上市，汨特石墨、湘菌科技、福星林业在天津股权交易所挂牌交易，全市共实现直接融资22.9亿元。

　　(二)狠抓项目建设，增强了发展后劲。

　　深入开展“项目建设与项目服务年”活动、“联手帮扶企业和产业发展升级”行动，预计全市亿元以上项目达到130个，完成投资198亿元。

　　产业项目扎实推进。预计完成投资431.1亿元。总投资57亿元的长岭炼油改扩建项目全面建成投产;巴陵石化化工改造建设项目完成投资31亿元，12万吨苯乙烯、10万吨双氧水装置建设、20万吨己内酰胺装置挖潜改造等子项目完工;东方雨虹二期、凯美特尾气回收、远大可建一期等重点项目建成投产;中国造纸装备自主化研制基地落户岳阳经济技术开发区;城陵矶临港产业新区签约项目30个，完成投资15亿元;汨罗“城市矿产”示范基地和以洞庭湖国际公馆为依托的湖滨综合旅游开发加快推进。

　　基础设施项目扎实推进。预计完成投资99.3亿元。随岳高速、S306华容段、进港路等建成通车;京港澳高速公路岳阳连接线昆山互通至收费站段、长炼路等提质改造工程完成;城陵矶临港产业新区长江大道等9条骨干道路基本拉通;通平和岳常等5条高速公路、炼化一体化公路、S207平江段、鸭栏码头一期、芙蓉大道北拓工程(湘阴段)等项目加快建设;改造农村公路1119公里。环洞庭湖基本农田重大建设一期第一年度工程全面竣工，湘江流域重金属污染治理项目进展顺利，铁山水资源保护和利用项目前期工作全面展开。钱粮湖、麻塘垸堤防加固、水库除险加固、灌区续建配套等一批水利项目加快实施，解决农村人口安全饮水30.3万人。全面启动农网升级改造，完成中心城区12.8万户居民智能化用电改造和金凤桥片区线路迁改。

　　城镇市政项目扎实推进。预计完成投资36亿元。启动岳阳市历史文化名城保护规划修编，完成环南湖总体城市设计;环南湖截污管网建设竣工;千亩湖旅游走廊开园;洞庭大道君山段、通海路、园艺路、云溪城南新区三路、冷水铺路三期等项目完成;南湖大道北段、云梦路完成油化提质;德胜北路完成主体工程;青年东路王家河大桥竣工通车;步步高城市综合体、天伦城金三角商业街、东茅岭步行街人防工程、天灯咀旧城改造等项目稳步推进;华容护城港综合治理、平江首家坪大道、岳阳县荣鹿公路、汨罗屈原南北路、湘阴太傅路延伸线、临湘向阳路拓宽改造等一批城建工程顺利实施。

　　(三)推进“五创提质”，美化了城乡环境。文明城市创建取得阶段性成果，我市城市公共文明指数测评进入全国地级市前30名，荣获“全国文明城市提名奖”，华容荣获“全国文明县城”称号。“环保创模”通过国家环保技术评估，全市共完成91个减排工程，二氧化硫、化学需氧量、氨氮分别削减3%、2.4%和4.1%;中心城区“退二进三”进展顺利，韶峰建材、新泰化肥等实现关停，中南科伦、同联药业启动新址建设。开展城乡环境卫生整洁行动，城镇生活垃圾无害化处理率达37%，全市“四清”、“四改”率分别达到10%和15%。城市环境综合整治定量考核获全省第一，湘阴评为“全省城乡环境卫生整洁行动十佳县”。“绿化创模”完成植树造林25.6万亩，森林覆盖率达45.3%;城区新增绿地110万平方米，人均公共绿地面积达9.3平方米，绿化覆盖率达39.8%。“治安创模”深入人心，新建电子眼614个，开展“打四黑、除四害”专项行动和“清网行动”，严厉打击涉黑、涉恶、涉赌、涉毒、涉黄等犯罪行为，社会治安综合治理考评继续保持全省先进。“交通创模”完成《城市交通管理规划》等方案，完善一批交通标线标牌，大力整治交通乱源，积极疏通交通拥堵路段，城区交通秩序逐步好转。中心城区加速提质，完成火车东站、邕园西路、岳阳大道沿线、金鹗东路、巴陵广场和南湖广场周边亮化改造，实施95条小街巷路面及设施改造，完成5条城区主干道建筑立面改造。“五创提质”由中心城区向县(市)区延伸，城乡面貌进一步改观，城市更加秀美宜居。

　　(四)大办惠民实事，改善了民生福祉。

　　完成41件省市实事任务，全市民生支出占财政一般预算支出71.7%。灾后重建全面完成。临湘、平江、岳阳县三个重灾区3517户因灾全倒户入住新居;修复水毁水利设施近5000处、耕地5.7万亩，补改种农作物9.9万亩，灾区交通、电力、通讯等基础设施全部恢复。国家的大力支持，社会各界的无私援助，灾区群众的艰苦奋斗，共同唱响了众志成城、重建家园的凯歌。社会就业持续增加。全市新增城镇就业人员5.5万人，新增农村劳动力转移就业8.6万人，零就业家庭实现动态清零;屈原启动全省统筹城乡就业示范区创建工作。保障性住房建设任务超额完成。积极争取国家支持，加大地方配套，共投入25.3亿元建设保障性住房，开工40397套，开工率113.5%，竣工36832套，竣工率103.5%。控物价保民生取得实效。认真落实国家稳定物价的各项政策措施，预计居民消费价格上涨5.1%;建立社会救助和保障标准与物价上涨挂钩的联动机制，向全市35.7万名低收入群众发放价格补贴4095万元。社会保障不断加强。城乡低保对象月人均补差分别达到182元和81元，大病医疗慈善救助基金规模达到5000万元、发放救助资金714万元，农村“五保”老人集中和分散供养标准继续提高，市儿童福利院投入使用，国泰阳光颐养苑即将投入运营。8个县(市)区纳入新型农村养老保险试点。在全省率先实现乡镇卫生院、社区卫生服务中心基本药物制度全覆盖，新农合参合率达到99.4%。城乡医疗条件进一步改善，市一医院东院、市中医院科教大楼投入使用，市二医院科教大楼加快建设，7家县(市)区人民医院、中医院完成改扩建。教育事业不断进步。完成166个校安工程建设;建成37所义务教育合格学校和19所农村公办幼儿园，市十五中顺利搬迁;岳阳中南工业学校和岳阳县职业中专被评为“国家首批职业教育改革示范校”;素质教育进一步加强。文化惠民受益面继续扩大。建成95个乡镇综合文化站和1585家农家书屋;送戏下乡1028场，公益电影放映4.4万场;城乡群众文化活动更加活跃，全民健身运动深入开展;《汨罗江畔的“城市矿产”》荣获“中国新闻奖”;全市图书馆、博物馆、文化馆、纪念馆全部免费开放;市档案馆晋升国家一级综合档案馆;市体育中心加快建设。扶贫开发力度加大。新一轮扶贫工作启动，帮扶贫困农民6.9万人，“雨露计划”培训贫困农民4170人。

　　(五)深化改革开放，提升了发展活力。

　　完成市属29家国有企业改制，妥善处理一批历史遗留问题。开展税收管理改革试点，推广二手房交易价格评税系统，推行稽查预告和“三自两免”制度，税收征管秩序进一步优化。成立湖南大湖传媒集团公司，组建市巴陵戏传承研究院，我市被评为全国文化体制改革先进地区和“扫黄打非”先进单位。启动市城市建设投资管理体制改革;下放经济社会管理权限，推进扩权强县;公立医院等事业单位改革稳步推进。对外开放不断扩大，预计全市实施内联引资项目561个，到位资金223.2亿元;实际利用外资1.86亿美元;进出口总额3.6亿美元。引进中粮集团、中建集团、中航集团、中种集团、中国铁建、中国水电八局、中纺集团、中轻集团、中联重科等一批央企名企落户。城陵矶至上海、宁波、太仓、南通四条“五定”始发班轮航线和城陵矶至香港、澳门直达航线正式开通，“重庆、武汉、长沙、上海”四地海关口岸通关模式在岳阳顺利实施，城陵矶保税港区申报工作正式启动。

　　(六)强化社会管理，促进了和谐稳定。

　　认真办理人民来信来访，努力化解各类矛盾纠纷。食品、药品安全监管得到加强。开展安全生产大排查、大整治，安全生产形势稳定。严厉打击传销，积极整治非法集资，市场秩序不断规范。进一步加强规划编制和管理，推进禁违拆违。试行城市干道项目化管理，推行“七位一体”路段综合管理模式，加强城区烟花鞭炮限放工作，推进数字城管建设和网格管理。计划生育工作实现保先进位和均衡发展，连续13年保持全省先进，华容、汨罗、湘阴、屈原、君山、云溪进入全省计划生育模范单位行列，全市人口自然增长率5.93‰。深入推进依法行政，建立监察、审计、法制联动协作监管机制，自觉接受人大法律监督和政协民主监督，全年承办人大议案、代表建议170件，承办政协建议案、委员提案474件。国防教育、国防动员、国防后备力量建设和人防工作继续加强，驻岳人民解放军、武警和民兵预备役部队服务经济社会发展的作用不断发挥。统计、外事、侨务、对台、广播、电视、新闻、出版、民族、宗教、国安、气象、史志、保密、老龄、残联、质监、检验检疫、驻外联络、住房公积金、移民、工会、共青团、妇联、科协、社会科学、邮政、通信、盐业、烟草等各项工作取得新进步。

　　各位代表，过去一年取得的成绩，是市委统揽全局、正确领导的结果，是市人大、市政协大力支持和监督的结果，凝聚着全市人民的心血和汗水。在此，我代表市人民政府，向全市各行各业的广大劳动群众，向各民主党派、工商联、无党派人士和各人民团体，向驻岳人民解放军、武警、消防部队指战员和政法干警，向所有关心、支持和参与岳阳改革建设的社会各界人士，表示崇高敬意和衷心感谢!

　　回顾一年的工作，我们也清醒地看到工作中存在的困难和不足。主要是：产业结构不尽合理，高耗能产业比重较大，新兴产业、旅游业、生态农业等比重偏小;大项目、好项目还不多，园区工业发展还不快，县域经济实力还不强;资金、土地、能源等生产要素制约较为突出;需要解决的民生诉求还比较多，维护社会和谐稳定的任务较为艰巨;经济发展环境有待进一步优化，公务员队伍建设有待进一步加强。对这些问题，我们将予以高度重视，采取有效措施，努力加以解决。

　　二、全面完成2012年各项工作任务

　　2012年，是本届政府任期的最后一年，是我市被国务院定为长江沿岸对外开放城市20周年。展望2012年，挑战与机遇同在，压力与动力并存，机遇大于挑战。从国际看，受国际金融危机特别是欧债危机影响，世界经济形势总体上仍将十分严峻复杂，但经济全球化深入发展的趋势没有改变，国际生产要素重新整合、产业布局优化调整，为我们扩大对外开放、特别是扩大引进带来新机遇。从国内看，我国经济虽然出现了一些新的阶段性特征，经济增长存在下行压力，物价上涨压力仍然较大，发展中不平衡、不协调、不可持续的矛盾和问题较为突出，但仍处于发展的重要战略机遇期，中央已确定“稳中求进”的工作总基调，实施积极的财政政策和稳健的货币政策，针对中小微型企业、战略性新兴产业、重大基础设施建设和民生等领域的信贷投放力度将进一步加大，中央重点保障、重点支持的领域正是岳阳需要加强、正在重点推进的领域，我们争取政策支持的潜力很大。同时，省委、省政府支持环洞庭湖生态经济圈建设和我市城陵矶港口平台建设、电煤储运基地建设、石化产业发展的力度进一步加大，有利于岳阳争取更多更好的发展条件。从我市看，全市经济发展转型升级、节能减排任务艰巨，部分企业生产经营困难，发展实体经济面临新的难题，但我们有历届市委市政府为岳阳发展打下的坚实基础，有社会大局和谐稳定、人民群众干事创业激情饱满的良好局面，岳阳的区位优势、新的竞争优势正在加速凸显。我们必须全面、正确研判形势，增强忧患意识，充分利用有利条件和积极因素，努力化解矛盾，更加周密地做好应对各种风险和挑战的准备，牢牢把握工作的主动权，奋力推动各项工作“上水平、争一流、作贡献”。

　　今年政府工作的指导思想是：全面贯彻落实中央、省市委经济工作会议及省市党代会精神，以科学发展观为统领，以“稳中求进、进中求强”为主调，抓好经济发展，创新社会管理，办好民生实事，维护社会稳定，加强自身建设，续写民本岳阳和谐崛起新篇章，夺取推进“四化两型”、建设“五市一极”新胜利。

　　主要预期目标是：地方生产总值增长12%以上;财政总收入增长15%以上，其中一般预算收入增长18%以上;固定资产投资增长23%以上;社会消费品零售总额增长17.5%以上;城镇居民人均可支配收入和农民人均纯收入分别增长11%以上和12%以上;城镇登记失业率控制在4.5%以内;居民消费价格上涨4%左右;人口自然增长率控制在6.8‰以内;省下达的节能减排约束性指标全面完成，各项社会事业协调发展。

　　(一)加快转型升级，推动经济更好更快发展

　　坚持新型工业化第一推动力不动摇，充分发挥岳阳经济技术开发区、城陵矶临港产业新区、县(市)区工业园区在推进新型工业化中的骨干作用，充分利用丰富的水土资源、优良的生态环境、深厚的文化底蕴和宝贵的江湖岸线等优势，加快滨湖示范区发展，推动环洞庭湖生态经济圈建设，实现绿色、低碳发展。

　　1、做好四篇文章，在更宽领域更高层次优化区域经济布局

　　以城陵矶临港产业新区为依托，打造湖南长江经济带。岳阳作为湖南唯一通江达海的港口城市，必须充分利用这一优势，打造一条属于岳阳、属于湖南的长江经济带。

　　发展港口经济。加强港区基础设施建设，按照“现代新港、产业新区、滨江新城、岳阳经济新增长极和长江中游区域性航运物流中心”的发展目标，在基本完成“三年搭骨架”的基础上，加快完善港区路网、管网、污水处理厂、标准化厂房等配套设施，启动城陵矶新港二期、铁路专用线和白杨湖综合配套服务区建设，加快实施长江航道疏浚工程，提高发展港口经济的承载力。加快发展航运物流业，启动湖南烟草进出口基地建设，加快建设港龙物流项目，依托大市场繁荣物流。积极引进船代、货代公司，实现江海联运直航常态化，积极推进对台直航。抓紧建立统一的港口信息平台，推动跨区域口岸合作。发挥“四关联动”优势，组织更多货物进出，力争集装箱吞吐量达到20万标箱。立足于引大引强，着力引进投资大、带动性强的战略投资者，做到落户一家企业、形成一个产业。启动创建城陵矶临港产业新区国家级高新技术产业开发区，推进城陵矶保税港区申报工作。科学规划、利用、管理好长江岸线资源，推进临湘鸭栏、云溪道仁矶、君山荆江门、华容塔市驿、岳阳县鹿角、屈原推山咀、汨罗营田港、湘阴漕溪港等沿江沿湖港口集群发展，引导一批大耗水、大运量产业沿江布局。积极发展船舶制造业，进一步支持岳阳纸业做大做强。

　　壮大石化产业。支持巴陵石化、长岭炼化进一步扩大炼油生产能力，尽快实现原油加工总量达到1500万吨的目标。重点发展炼油产品综合利用、己内酰胺与环己酮、特种环氧树脂、高档锂系聚合物、炼油化工催化剂等优势产业，支持巴陵石化建设世界一流的非乙烯精细化工企业。围绕炼化一体化，做大配套产业，大力开展关联地区、关联产业、关联企业招商，促进长岭炼化、巴陵石化产品本地终端化。发展云溪精细化工园、长炼工业园、儒溪化工园等石化产业园区，推动全市石化产业产值向2000亿元迈进。

　　建设湘北能源基地。支持华能岳阳电厂扩大产能、华能供热改造及配套管网项目建设。充分发挥江海联运和交通枢纽优势，开辟电煤运输新通道，建设湖南煤炭储备(中转)基地。推进岳阳县鹿角、临湘黄盖湖、君山三江口等沿江风力发电项目建设。继续推进小墨山核电项目前期工作。

　　以岳阳经济技术开发区为依托，发展战略性新兴产业。进一步提升产业发展规划，引进一批拥有核心技术、创新能力强的战略项目，把岳阳经开区打造成对外开放的窗口、体制创新的试点、产业升级的平台、城乡统筹的示范、“两型”建设的领头雁。

　　提升发展先进装备制造产业。以中科电气、科美达为重点，建设国家磁力设备检验检测中心和国家电磁产品设计与研发中心，规范行业约束，促进良性竞争，加快发展电磁冶金成套设备产业。以国泰机械为重点，加快发展造纸机械设备产业。以雷博电气为重点，加快发展特种电机产业。

　　聚集发展生物医药产业。支持中南科伦和同联药业新厂建设。推进中小医药企业与大企业协作配套，延伸产业链条，推动全市生物医药产业产值向百亿元迈进。　培育发展电子光伏产业。加快卫星导航产业发展，大力支持国信军创建设卫星导航产业岳阳基地。推进田谷电子信息产业园做大规模，培育一批领军企业。支持湖南桑乐数字化太阳能热水器升级扩张，支持千盟电子、天元电子等企业加快发展。

　　扶植发展设计创意产业。支持百利科技等企业拓展市场，支持君山科技印刷工业园扩规提质，推动文化创意产业园区和基地建设。发展新媒体、数字技术、视觉艺术创作、工业产品设计等新型业态，构建特色文化创意产业体系。

　　以岳阳楼君山岛5A级景区为依托，建设洞庭湖旅游度假区。充分发挥和放大5A级景区的品牌效应，整合旅游资源，加快产品开发，推进国家级洞庭湖旅游度假区创建工作，提升旅游产业的整体水平和带动能力。

　　拓展岳阳楼君山岛景区。抓好文庙、慈氏塔、君山野生荷花世界、濠河生态湿地、虹宇生态农庄等景点建设，与岳阳楼君山岛景区整体打捆、统一营销，丰富5A级景区内涵，提升竞争力。

　　开发环南湖旅游。按照科学规划、建设、开发、保护和管理的思路，抓好环南湖城市设计整体策划，完善总体规划，启动一批分项规划建设，构建生态文化新景区、现代滨湖新城区、旅游休闲度假区。

　　做强县域特色旅游。突出区域特色，发挥比较优势，大力发展平江红色游和体验游、汨罗屈子文化游、湘阴休闲游、张谷英民俗游、临湘和华容山水游。加快推进洞庭湖国际公馆、洋沙湖度假村、任弼时纪念馆二期工程、石牛寨国家地质公园等一批重点旅游项目。

　　推进旅游与文化融合。以文化提升旅游品位，打造旅游品牌，加快建设屈子文化园、左宗棠纪念园，启动旅游实景剧项目。办好君山爱情文化节、端午旅游文化节、洞庭湖国际观鸟节等活动，促进巴陵戏、长乐故事会等非物质文化遗产保护传承与旅游相结合，发展工业旅游、农业休闲旅游、商贸旅游，不断丰富旅游新业态。

　　以城镇和园区为依托，提升县域经济综合实力。进一步扩权强县，加速新型城镇化、工业园区和农业现代化发展，以城带乡、以工促农，提高县域经济发展水平，努力实现财政收入上台阶。

　　推进新型城镇化。科学规划，合理布局，统筹城乡基础设施建设、生态环境保护和公共服务，以县级市、县城和中心镇为重要节点，构建县城支撑县域经济、中心镇带动乡村发展的新型城镇体系。突出对接长株潭，推进湘阴、汨罗、屈原、平江四地连片开发、组团发展;发挥近城优势，支持岳阳县北靠、与市区融合发展;立足省际边界区域优势，推动临湘、华容提速发展;加大对沿江、沿路、沿边和特色城镇支持力度，建设一批精品小城镇。因地制宜，凸显特色和个性，加强乡村规划，推进乡村居民点集中布局，逐步形成富有地域特色的村庄体系。

　　壮大特色工业园区。以“两型园区”发展为主线，以引进科技含量高、可持续发展能力强的龙头企业为重点，以特色谋求竞争优势，形成园区规模集聚效应。深入实施“千百十”工程，引导工业企业向园区集中，推进平江汽车和工程机械零部件基地、岳阳县食品轻工和生物医药工业集中区、华容医药纺织和电子光伏产业园、湘阴先进制造和电子信息产业园、临湘建材矿产和化工农药工业园、汨罗“城市矿产”基地等园区建设，构建特色鲜明、功能完善、分工协作的产业园区体系。以园区为载体，大力发展配套产业和企业，增强县域承载能力和吸纳能力，辐射带动县域经济全面发展。

　　提高农业现代化水平。全面落实强农惠农富农政策，健全农村发展体制机制，促进农业增产、农民增收、农村发展。加快农业科技进步和农业技术推广体系建设，推进农业机械化，提高农业物质技术装备水平。抢抓国家大兴水利建设的机遇，建设一批水利工程，新建一批高标准农田，提高农业综合生产能力和防灾减灾水平。严格落实耕地保护制度，做好种子、化肥、农药等重要农资的储备供应，毫不放松地抓好粮食生产，切实保护农民种粮积极性，抓好“菜篮子”工程建设。发展畜禽、茶叶、油料、蔬菜、水产和棉麻等特色优势产业，开发林下经济，推进农业标准化生产、规模化经营和集约化发展，提高农产品产量和质量。实施农产品加工业振兴计划，壮大一批农产品加工龙头企业。支持屈原国家级现代农业示范区、岳阳县台湾农民创业园、湘阴和华容全国农业标准化示范县、君山全国农产品加工示范基地、临湘楠竹产业园等农业园区建设，支持以君山银针为代表的黄茶产业和临湘黑茶产业发展，提升农业产业化水平，引领现代农业发展。坚持龙头带动，特色发展，打造食品千亿产业集群。进一步健全农民专业合作社、农产品流通中介组织、农产品质量检查和认证、农业信息等农业产业化社会服务体系。大力发展农业职业教育，着力培育“有文化、懂技术、善管理、会经营”的新型农民，全面提升农民素质。深入开展“万企联村、共同发展”活动，实施“百城千镇万村”工程和“示范村庄”建设，着力加强农村基础设施建设和公共服务，抓好饮水安全、道路建设、电网改造和农村危旧房改造，改善农村人居环境和生产条件，深入推进社会主义新农村建设。进一步加大扶贫开发力度，促进专项扶贫与行业扶贫、社会扶贫相结合，形成扶贫攻坚的强大合力，坚持不懈地消除贫困落后，让困难群众早日过上富裕安康的生活。

　　2、实施三大攻坚，以更大气魄更硬措施推动发展提速提质

　　实施项目引进和建设攻坚行动。加快经济发展，关键靠项目支撑。努力引进一批大项目、好项目，掀起项目建设新热潮。重点推进100个重大项目建设，力争完成投资196.6亿元。主要是：长岭炼化1150万吨炼油综合能力配套项目和环氧丙烷及配套双氧水项目、巴陵石化350万吨炼油装置改造、10万吨乙酸仲丁酯生产项目、远大可持续建筑低碳经济科技园、压力传感器产业化项目、澳门名嘉城市商业综合体等30个产业项目;京港澳高速公路复线、环洞庭湖基本农田重大建设工程、天灯咀旧城改造，平江、岳阳县、临湘路网工程等20个基础设施项目;洞庭湖综合治理、湘江流域重金属污染治理、汨罗江及支流综合治理等20个生态环保项目;岳阳楼区居民安置房建设、农村饮水安全、华容长江引水工程、新一轮城区和农村电网改造等30个民生工程项目。围绕重点项目组织开展项目建设竞赛活动，推行一个重点项目、一位市县级领导、一个责任单位、一名责任人、一套方案、一支队伍的项目服务模式。

　　实施淘汰落后产能攻坚行动。淘汰落后产能是转变经济发展方式、推进新型工业化和实现包容性增长的必然要求。坚决依法关闭落后产能企业，全面完成际华3517等首批6家重点排污企业“退二进三”、“退城进园”，启动第二批企业“退二进三”、“退城进园”，关停一批“十五小”企业。充分发挥科技对产业升级的支撑作用，运用高新技术和先进适用技术，对落后产能进行改造，扎实推进燃煤锅炉改造等重点节能减排项目，加强清洁生产示范试点建设，扩大企业清洁生产，促进企业低耗、高效发展。严格市场准入，推行新上项目节能评估和“区域限批”制度，强化安全、环保、能耗、物耗、质量、土地等指标的约束作用，鼓励发展低消耗、低污染的先进产能，防止新增落后产能。强化企业的主体责任，推动化工、陶瓷、水泥、冶炼、发电和造纸等重点行业节能降耗。建立健全淘汰落后产能长效机制，实行年初公布淘汰落后产能企业名单，分季调度，年终考核验收，确保淘汰落后产能工作取得明显成效。

　　实施城乡环境整治攻坚行动。在全市所有乡镇开展“四清、四改”整治行动，推行“户分类、村收集、乡中转、县处理”的农村垃圾分类处理模式，创建300个市级新农村建设清洁家园样板村。集中整治中心城镇、旅游景区、交通要道沿线和城郊结合部环境。推广生态种养，深化畜禽污染防治，控制化肥、农药污染，切实消除农业面源污染。在全市开展“集中整治年”活动，依法整治矿产资源、河道砂石资源开采秩序，严厉打击滥采滥挖的不法行为。切实加大铁山水库等重点水资源环境整治力度，保护水环境。深入开展城乡绿化，大力实施沿水、沿路和城镇周边绿化工程，全力推进“身边增绿”，扎实推进园林式单位、森林乡镇、生态村庄和绿色校园创建活动，全年完成造林20万亩以上，新增城区绿地面积170万平方米以上。

　　3、深化“五创提质”，以更高品位更强活力建设宜居城市

　　加强规划管控。坚持中心城区“东扩、西连、南延、北靠、中提”发展战略，突出山水生态保护，抓好环南湖、东风湖、芭蕉湖、吉家湖等滨水地段城市设计，加快编制产业、环保、交通、管网等专项规划。强化规划约束，严格规划执法，严禁违法建设，全面开展禁违拆违集中整治行动，严肃查处涉规的违法违纪案件。以铁的手腕、铁的纪律、铁的措施，严厉打击、坚决遏制违法建设行为。

　　加快扩容提质。进一步拉开城市发展大骨架，高标准建设京港澳高速公路岳阳连接线提质改造工程，启动临湖路、奇康路建设，推进“跨湖发展”。创新理念，拓宽融资渠道，启动洞庭新城建设;积极对接，加强与国家开发银行合作，加快金凤桥片区开发。启动城区河湖连通环城水系工程，搞好环南湖水环境综合治理，完成王家河流域综合治理一期工程，完成32条中心城区主次干道“白改黑”任务，完善城区小游园、人行天桥、公厕等便民设施建设，改善人居环境，增强服务功能，提高城市品位。

　　提升城管水平。全市动员，全民参与，全面开展新一轮全国文明城市创建活动，力争“环保创模”成功夺牌、“绿化创模”和“交通创模”顺利通过国家验收，深入推进“治安创模”。运用“数字城管”系统，推进城市管理信息化、标准化、网格化。加大城区烟花鞭炮限放力度。注重公益性、服务性，进一步理顺殡葬管理体制，规范殡葬服务市场秩序。深入开展爱国卫生运动，确保顺利通过国家卫生城市复查。加强交通管理，提高市民文明交通意识，优化路网结构，优先发展公共交通，规范客运市场秩序，合理规划建设停车场地，发展智能交通系统，有效化解中心城区日益凸显的“停车难、行车难”问题，为市民出行方便和安全创造良好条件。

　　活跃城市经济。努力增加城乡居民特别是低收入群众收入，提高中等收入者比重，提高居民消费能力，积极扩大居民消费。改善消费环境，抓好市场监测和供应，加强市场经营监管，深入推进消费安全工程，维护消费者合法权益。加快消费结构升级，积极培育文化、信息、旅游等消费热点，发展电子商务、网上购物、在线交易等消费方式，拓展服务类消费。培育主力商圈和商业综合体，建设胥家桥市场集群，完成中心城区农贸市场改造提质任务。培育家政服务、养老服务、健康服务等新型服务业态，引导房地产业健康理性发展。大力发展非公有制经济，降低准入门槛，鼓励充分竞争，凡是政策和法律没有禁止的领域，都允许各类资本进入。重点支持创新型、创业型、劳动密集型中小微型企业发展，认真落实国家结构性减税政策，进一步完善服务体系。

　　4、深化改革扩大开放，以更新理念更广视野破解发展难题

　　坚持不懈改革创新。深化国有企业改革，妥善处理国企改制遗留问题，尽快完成改制扫尾工作;加强现代企业制度建设，改进国企资产监管，确保国有资产保值增值。推进水利改革，完善防汛抗旱减灾机制，实行最严格的水资源管理制度，抓好水利工程管理体制、基层水利服务体系等改革。深化财税管理体制改革，调整和完善中心城区财政体制。抓好投融资体制改革，优化财政投资运行机制，确保投向民生领域的财力稳步增长;举办金融博览会，促进“银、保、证、企”对接互动;全力支持企业上市、发行债券;加强与天津股权交易所合作，力争设立华中股权交易中心;加快组建岳阳市农村商业银行，积极引进股份制商业银行，引进设立村镇银行和小额贷款公司，努力缓解中小微型企业融资难问题;依法打击恶意逃废银行债务、非法集资和高利贷行为，优化金融生态。增强科技创新能力，健全覆盖全市主导产业的产学研结合创新体系，新组建一批省级以上创新平台，实施一批关键共性技术攻关和重大科技成果转化项目，争取专利授权量突破1000件、具有自主知识产权的高新技术产品达到600种。大力实施商标战略，推动品牌创建。

　　坚持不渝扩大开放。牢固树立“开放兴市”理念，充分发挥口岸优势，大力发展开放型经济。突出招商引资，继续推进“招商引资和项目建设年”活动，积极承接产业转移，对接央企名企，着力招大引强。依托国家级平台和大厂优势，做大做强产业集群。加强中介招商、驻点招商和小分队招商，提高招商实效。加强区域合作，实施“走出去”战略，发挥地缘、人缘优势，加强与珠三角的经贸交流;发挥通江达海的航运物流优势，加强与长三角的对接协作。扩大对外贸易，发展加工贸易，进一步扩大石化产品出口，继续保持机电、轻纺产品、农副产品、工艺产品等传统产品的出口优势。加强引才引智，积极引进国际金融、港口发展、环保旅游、城建规划、企业经营管理等领域的专业人才。

　　坚定不移优化经济发展环境。建立健全以政府信用为关键、企业信用为重点、个人信用为基础的社会信用体系。加强机关效能建设，继续推进行政审批职能相对集中改革，进一步优化办事流程，实行一个窗口对外、一站式审批、“一票制”收费、“一条龙”服务，提高服务质量和效率。大力推行企业“宁静日”制度和“围墙法则”，建立项目周边环境责任制度。推进政务公开，加强电子政务建设，完善公共资源交易平台。像爱护生态环境一样爱护经济发展环境，像抓项目一样抓经济发展环境，深入开展“机关作风大家评”活动，完善优化经济环境的考核讲评办法，加大奖惩力度，对损害投资创业者合法利益、扰乱市场秩序等破坏经济发展环境的人和事，严厉查处，决不姑息。

　　(二)加强和创新社会管理，建设和谐美好家园

　　提高社会管理科学化水平，努力形成社会管理人人参与、和谐社会人人共享的生动局面。

　　推进法治建设。认真实施《法治湖南建设纲要》，全面推进依法治市。切实加强法制宣传教育，推进“六五”普法和“法律六进”活动，营造全社会自觉学法守法用法的良好氛围。加强民主政治建设，自觉接受人大依法监督和政协民主监督;扩大基层民主，健全城乡基层群众自治机制，加强基层依法治理，畅通社情民意表达渠道，切实保障人民群众的知情权、参与权、表达权和监督权。坚决维护社会公平正义、维护社会公共秩序，依法规范信访行为，依法严处违法信访行为，依法调处各类矛盾纠纷，加强社会治安综合治理，坚决扫黑除恶，严惩黄赌毒等违法犯罪行为，让全市人民安居乐业。

　　强化公共管理。强化安全生产监管，深入开展“安全生产年”活动，推进安全生产标准化，全面落实安全生产责任制，深化非煤矿山、道路交通、水上交通、危险化学品、特种设备、烟花爆竹等重点专项整治，确保安全生产形势持续稳定向好。强化应急管理，加强公共安全体系建设，完善自然灾害、事故灾难、公共卫生事件、社会安全事件的监测预警和应急处置机制，健全应急指挥体系和应急救援队伍，提高应急救援和应急处置能力。建立完善互联网动态管控、信息巡查、舆情监测机制，启动互联网经营场所监管平台建设，加强虚拟社会管理。创新警务管理，优化整合警务力量，加大警力向治安一线下沉力度，建立便民利民处警机制，提升快速处置能力。强化食品药品安全监管，加强食品药品生产、经营和餐饮安全各环节管理，严厉打击食品药品违法犯罪，让人民群众吃上放心菜、放心油、放心肉和放心药。加强人口和计划生育工作，突出“阳光计生建设年”主题，进一步完善“依法管理、村(居)民自治、优质服务、政策推动、综合治理”工作机制，严厉整治违法生育和“两非”行为，稳定低生育水平，提高出生人口素质。

　　加强社区建设。以构建和谐社区为目标，推动城市和农村社区工作健康发展。建立财政支持、社会赞助、费随事转、有偿服务等多元筹资机制，加大社区基础设施建设投入，按照便民、利民、惠民的原则，逐步完善社区服务窗口、生活配套设施、休闲娱乐设施，提高社区服务功能。进一步加强社区工作队伍建设，配齐配强社区工作人员，保障社区工作人员工资福利待遇，加强业务培训，提高队伍整体素质。创新社区管理体制，探索社区扁平化管理，在城区进行区直管社区试点。推进农村社区建设，积极探索农村社区管理有效途径和办法。

　　(三)大力改善民生，让人民群众共享发展成果

　　坚持把保障和改善民生作为政府一切工作的出发点和落脚点，围绕群众最关心、最直接、最现实的利益问题，集中力量办好民生实事，全面完成省市实事任务，年底向全市人民交账，让更多改革发展成果惠及广大人民群众，提升人民群众的幸福感和满意度。

　　扩大城乡就业。以创业带动就业，大力支持实体经济发展，鼓励全民创业，积极创建省级创业型城市，着力推进屈原管理区创建全省统筹城乡就业示范区。搭建对外劳务合作服务平台，大力推动劳务对外输出，拓宽就业渠道。全年新增城镇就业4.6万人以上，新增农村劳动力转移就业4万人以上，城镇“零就业”家庭继续动态清零。

　　加强社会保障。强化价格调控，努力保持价格总水平基本稳定，认真实施社会救助和保障标准与物价上涨挂钩的联动机制。抓好保障性住房建设，推进中心城区旧城、城中村、筒子楼、棚户区改造，完成保障性安居工程和各类棚户区改造4.96万套，努力解决城市中、低收入家庭住房困难。扩大社保“五大保险”覆盖面，推进基本养老保险城乡全覆盖。提高低保、五保、优抚、孤儿等保障标准。实施重度残疾人特殊生活补贴、部分辅助器具免费配送、6岁以下贫困残疾儿童抢救性康复等惠残助残项目。开展大病医疗救助，进一步搞好慈善基金募集和管理工作。

　　推进公共卫生服务。发展医疗卫生事业，建立健全城乡基本公共卫生服务网络，普及基本公共卫生均等化服务。启动市妇幼保健院和市儿童医院整体搬迁，实施省安康医院、8家县级卫生监督中心和5家乡镇中心卫生院建设。新农合参合率巩固90%以上，最高支付限额由8万元提高到15万元。落实儿童先天性心脏病、白血病、农村五保对象免费治疗政策，推广单病种费用包干政策。医疗卫生事业关系人民群众健康和家庭幸福，我们要克服一切困难，把这项事业办好。

　　发展教育事业。加大教育投入。优化中小学校布局，促进义务教育均衡发展。坚持普、职合理分流，加强职业教育专业规范化建设。提高高等教育质量，培养适应社会需求的各类人才。推进学前教育三年行动计划。坚持德育为先、能力为重，全面推进素质教育。加强教师队伍建设，继续抓好校安工程，加强校舍、校车、校园食品安全监管。教育不普及不提高，就不可能富民强市，这个道理我们永远要铭记。　建设文化强市。坚持以社会主义核心价值体系引领文化发展，加强机关、学校、企业文化建设，不断提高全体市民精神文明素质。深化文化体制改革，推进政企分开、政事分开、管办分离，加强国有文化资产管理，推进公益性国有文化单位劳动就业、人事人才、收入分配和社会保障制度改革，激活内力，强化服务，提高水平。推动公益性文化事业发展，加大公共文化基础设施建设力度，加快市广电中心、市体育中心、市青少年活动中心、市档案馆新馆、市图书馆新馆、洞庭湖博物馆等设施建设。按照“强化主体、做大项目、创新业态、拓展市场”的要求，推动文化产业跨越式发展。加强社区和农村文化建设，合理配置城乡文化资源，实施以城带乡的文化帮扶计划，建立一批基层文化工作阵地。继续实施“演艺惠民，送戏下乡”工程，推进文化惠民，繁荣文艺创作，满足人民群众精神文化需求。加强文化人才队伍建设，建立专业文化人才储备库，制定培养、引进专业人才的特殊政策，重视青年文化人才，完善文化人才激励机制。大力开展全民健身运动，发展体育事业。

　　继续加强国防教育、国防动员、民兵预备役和人防工作，搞好拥军优属、拥政爱民，促进国防建设与经济建设良性互动。切实抓好民族、宗教、史志、妇女、儿童和老龄等工作，推进各项社会事业协调发展、全面进步。

　　三、以过硬的自身建设保障工作落实

　　注重民主、担当、实干、公廉，努力建设法治政府、服务政府、责任政府和廉洁政府。

　　坚持科学施政。加快岳阳发展是全市人民的企盼，是政府工作的第一要务。坚持群众路线，大兴调查研究之风，问政于民，问计于民，问需于民。健全集体决策制度，实行科学民主决策，尊重经济社会发展规律，努力创造经得起实践、人民和历史检验的实绩。下决心精简会议，精简文件，控制检查，减少应酬，把心思用在谋发展上，把激情用在干事业上，把精力用在抓工作上。

　　坚持依法行政。认真贯彻实施《湖南省行政程序规定》和《湖南省政府服务规定》，严格按照“职权法定、权责统一”的要求，进一步规范行政权力运行，提高政府执行力和公信力。落实行政决策听证制度，完善行政决策配套制度，建立重大行政决策风险评估机制;健全行政决策智力支持系统，进一步完善法律顾问工作制度;严格执行规范性文件“三统一”制度、备案审查制度、有效期制度、定期清理制度、审查申请制度等，认真落实行政裁量权基准制度和行政执法案例指导制度，加强行政复议规范化建设。依法履行各项职能，按照法定权限办事;树立程序意识，按照法定程序办事;改进执法方式，规范行政执法，严格落实执法责任制，营造良好的政务环境。

　　坚持从严治政。强化行政效能监察，健全重大决策部署执行情况专项督查制度，坚决纠正和严肃查处上有政策、下有对策，有法不依、有令不行、有禁不止的现象和问题，确保政令畅通。加强对事关全局工作的大事、改善民生的实事、久拖不决的难事的督查，推动工作落实。强化政府行政绩效考核作用，完善绩效考核办法，加强履行职能职责考核，搞好绩效考核结果运用。坚持精细化服务管理，追求零差错、零失误、零事故。严格工作责任，坚持有错无为问责，努力建设一支政治强、素质高、业务精、作风好的公务员队伍。

　　坚持廉洁从政。加强党风廉政建设，严格遵守廉政准则，认真落实“一岗双责”，以更加坚定的决心、更加有力的举措推进惩治和预防腐败体系建设。建立健全决策、执行、监督相对分离、相互制约的行政运行机制，严惩公权滥用。强化公务用车购置及运行、公务招待、因公出国(境)、公职人员招录和公共资源交易监管，加强对工程建设、国土资源、规划许可、药品器械采购等重点领域和关键环节的监管，加强对各类基金和专项资金的监管。厉行节约，严格控制各类论坛、节会、表彰等活动，降低行政运行成本，推动廉洁透明政府建设。

　　各位代表!困难和挑战考验着我们，机遇和期待召唤着我们，责任和使命激励着我们。让我们在中共岳阳市委的坚强领导下，凝聚全市人民的智慧和力量，坚定信心、抢抓机遇、开拓创新，强力推进“四化两型”，加快建设“五市一极”，为续写民本岳阳和谐崛起新篇章而努力奋斗，以优异成绩向党的十八大献礼!
